# Noninvasive detection of tumor-associated mutations from circulating cell-free DNA in hepatocellular carcinoma patients by targeted deep sequencing

**DOI:** 10.18632/oncotarget.9629

**Published:** 2016-05-26

**Authors:** Wenjun Liao, Huayu Yang, Haifeng Xu, Yanan Wang, Penglei Ge, Jinjun Ren, Wei Xu, Xin Lu, Xinting Sang, Shouxian Zhong, Hongbing Zhang, Yilei Mao

**Affiliations:** ^1^ Department of Liver Surgery, Peking Union Medical College (PUMC) Hospital, PUMC & Chinese Academy of Medical Sciences, Beijing, China; ^2^ State Key Laboratory of Medical Molecular Biology, Department of Physiology, Collaborative Innovation Center for Cancer Medicine, Institute of Basic Medical Sciences and School of Basic Medicine, PUMC & Chinese Academy of Medical Sciences, Beijing, China

**Keywords:** circulating cell-free DNA, circulating tumor DNA, hepatocellular carcinoma, tumor-associated mutation, MiSeq sequencing

## Abstract

**Background:**

Detection of circulating cell-free DNA (cfDNA) has potential clinical value for assessing tumor biology in patients with hepatocellular carcinoma (HCC), yet many traditional assays lack robustness. This study was the first to apply a high-throughput sequencing platform to detect tumor-associated mutations in HCC from circulating tumor-derived DNA (ctDNA) and to evaluate the utility and feasibility of this approach.

**Methods:**

Using the MiSeq™ system, plasma and matched tumor DNA samples were analyzed for hotspot mutations in the *TERT*, *CTNNB1*, and TP53 genes that had been verified as the most prevalent mutations in HCC. We compared tumor and plasma data and prospectively investigated the association between significant mutations detected in ctDNA and the patients' clinical outcomes.

**Results:**

In 41 patients, we detected tumor-associated mutations for HCC in 8 (19.5%) plasma samples. Among them, one showed a tumor-associated mutation in ctDNA but not in the tumor tissue which we used to detect. We also found that ctDNA with mutations could be detected more easily in patients who suffered vascular invasion (P=0.041) and predicted a shorter recurrence-free survival time (P<0.001). There was no relationship between detectable mutations and concentration of cfDNA (P=0.818).

**Conclusions:**

The results of our study suggest that tumor-associated mutations detected in plasma are associated with vascular invasion and might be used to predict a shorter recurrence-free survival time for HCC patients. This kind of biomarker can overcome the limitations of tumor heterogeneity. Moreover, the diagnostic performance is improved if multiple mutations in different genes are combined.

## INTRODUCTION

Circulating cell-free DNA (cfDNA) is a type of cell-free nucleic acid (cfNA) that is predominantly derived from apoptotic and necrotic cells but is also released by living eukaryotic cells [1, 2]. Evaluation of this naturally occurring biological material might have potential clinical application for the detection and surveillance of major cancers because abnormal forms of tumor-derived cfDNA (ctDNA) are more likely to be present in these patients [1–3]. However, circulating cfDNA is present in only a few thousand amplifiable copies per milliliter of blood and typically contains fewer than 180 base pairs (bp), of which only 1% may be ctDNA and diagnostically relevant [4–6]. This places crucial limitations on the use of circulating cfDNA assays involving quantitative analyses, as well as most qualitative analyses.

The development of specialized techniques with high analytical sensitivity now allows reliable detection of tumor-specific genetic mutations present at frequencies as low as 0.01% [7, 8]. The application of this kind of digital genomic assay that allows discrimination of rare mutant variants in ctDNA has been reported in a wide range of cancers: for example, noninvasive detection of epidermal growth factor receptor (*EGFR*)- mutants in lung cancer [9] and phosphatidylinositol-4,5-bisphosphate 3-kinase, catalytic subunit alpha (*PIK3CA*)-mutants in breast cancer [10], and many other kinds of genetic-mutants in corresponding tumors. However, mutation detection in plasma DNA as a “liquid biopsy” has rarely been applied to hepatocellular carcinoma (HCC), even though it is the fifth most common cause of cancer in the world [11] and the mortality rate is rapidly increasing. Recent studies using whole-exome sequencing have revealed a global picture of the molecular genetics of HCC, and mutations in the telomerase reverse transcriptase (*TERT*), tumor protein p53 (*TP53*), and catenin beta 1 (*CTNNB1*) genes have emerged as the most prevalent alterations [12–14]. Therefore, targeting these specific frequent genetic aberrations in ctDNA might possibly be used to detect and assess HCC.

In the current study, we applied a high-throughput sequencing platform, Illumina MiSeq, for noninvasive detection of rare mutations in circulating cfDNA from plasma of HCC patients for the first time. We were interested in determining whether such an approach could be used to provide utility information for personalized medicine.

## RESULTS

### Patient characteristics

Between December 2013 and August 2014, a total of 41 patients with primary HCC were included in this study. All patients agreed to undergo a cfDNA assay on plasma obtained before surgery. The clinical characteristics of study patients are presented in Table 1. We also displayed the detailed information about the prior-treatment status of 41 cancer patients in Supplementary Table 1. In addition, 10 volunteers without HCC consented to participate in this study as a control group and donated peripheral blood after the informed consent was signed. Among these, four were healthy volunteers and the other six had been diagnosed with hepatic hemangioma, hepatic cystic echinococcosis, focal nodular hyperplasia, epithelioid hemangioendothelioma, and intrahepatic cholangiocellular carcinoma, respectively. None of the control subjects had chronic hepatitis or cirrhosis. The somatic mutation status that was detected in these volunteers is presented in Supplementary Table 2.

**Table 1:** Correlation between tumor-associated mutations and clinicopathological parameters

Clinical Characteristic		Patients (n=41)		T-TERT (N=29)	T-TP53 (N=27)	T-CTNNB1 (N=11)	Any T (N=39)	P-TERT (N=2)	P-TP53 (N=2)	P-CTNNB1 (N=4)	Any P (N=8)
		No.	%								
**Age:**	≤60 >60 P	25 16	61 39 -	19 10 0.698	15 12 0.657	6 5 0.739	24 15 0.959	1 1 1.000	2 0 0.522	2 2 1.000	5 3 1.000
**Gender:**	Female Male P	8 33	19.5 80.5 -	4 25 0.749	4 23 0.751	2 9 1.000	7 32 0.858	1 1 0.379	0 2 1.000	1 3 1.000	2 6 0.659
**Alcohol:**	Yes No P	14 27	34.1 65.9 -	11 18 0.745	10 17 0.807	4 7 1.000	13 26 0.939	0 2 1.000	2 0 0.133	1 3 1.000	3 5 1.000
**Cirrhosis:**	Yes No P	24 17	58.5 41.5 -	14 15 0.396	16 11 0.953	7 4 0.760	23 16 0.968	2 0 0.511	2 0 0.511	2 2 1.000	6 2 0.458
**HBV:**	Yes No P	38 3	92.7% 7.3% -	28 1 0.637	25 2 1.000	10 1 1.000	36 3 1.000	2 0 1.000	2 0 1.000	3 1 0.320	7 1 0.522
**Tumor size:**	<5cm ≥5cm P	24 17	58.5 41.5 -	16 13 0.779	17 10 0.715	7 4 0.760	23 16 0.968	1 1 1.000	1 1 1.000	3 2 1.000	5 3 1.000
**Vascular invasion**	Yes No P	25 16	61 39 -	20 9 0.492	16 11 0.887	4 7 0.182	24 15 0.959	2 0 0.522	2 0 0.522	4 0 0.281	8 0 0.041*
**Differentiation**	Well Moderate Poor P	9 22 10	21.9 53.7 24.4 -	3 17 9 0.482	2 20 5 0.191	5 4 2 0.333	9 21 9 0.987	0 1 1 1.000	0 2 0 1.000	2 1 1 0.533	2 4 2 1.000
**Preoperative AFP**	>20 ng ml^−1^ ≤20 ng ml^−1^ P	26 15	63.4 36.6 -	20 9 0.630	17 10 0.970	5 6 0.318	25 14 0.949	2 0 0.535	2 0 0.535	2 2 0.626	6 2 0.696
**Tumor number**	Single Multiple P	30 11	73.2 26.8 -	19 10 0.491	19 8 0.801	8 3 1.000	28 11 0.890	1 1 0.485	1 1 0.485	4 0 0.558	6 2 1.000

Abbreviations: T-TERT, TERT mutation status in tumor tissue DNA; T-TP53, TP53 mutation status in tumor tissue DNA; T-CTNNB1, CTNNB1 mutation status in tumor tissue DNA; Any T, all of these mutations in tumor tissue DNA; P-TERT, TERT mutation status in Plasma DNA; P-TP53, TP53 mutation status in Plasma DNA; P-CTNNB1: CTNNB1 mutation status in Plasma DNA; Any P, all of these mutations in Plasma DNA

### Mutation analysis of circulating cell-free DNA and matched primary tumor DNA

**Figure 1:**
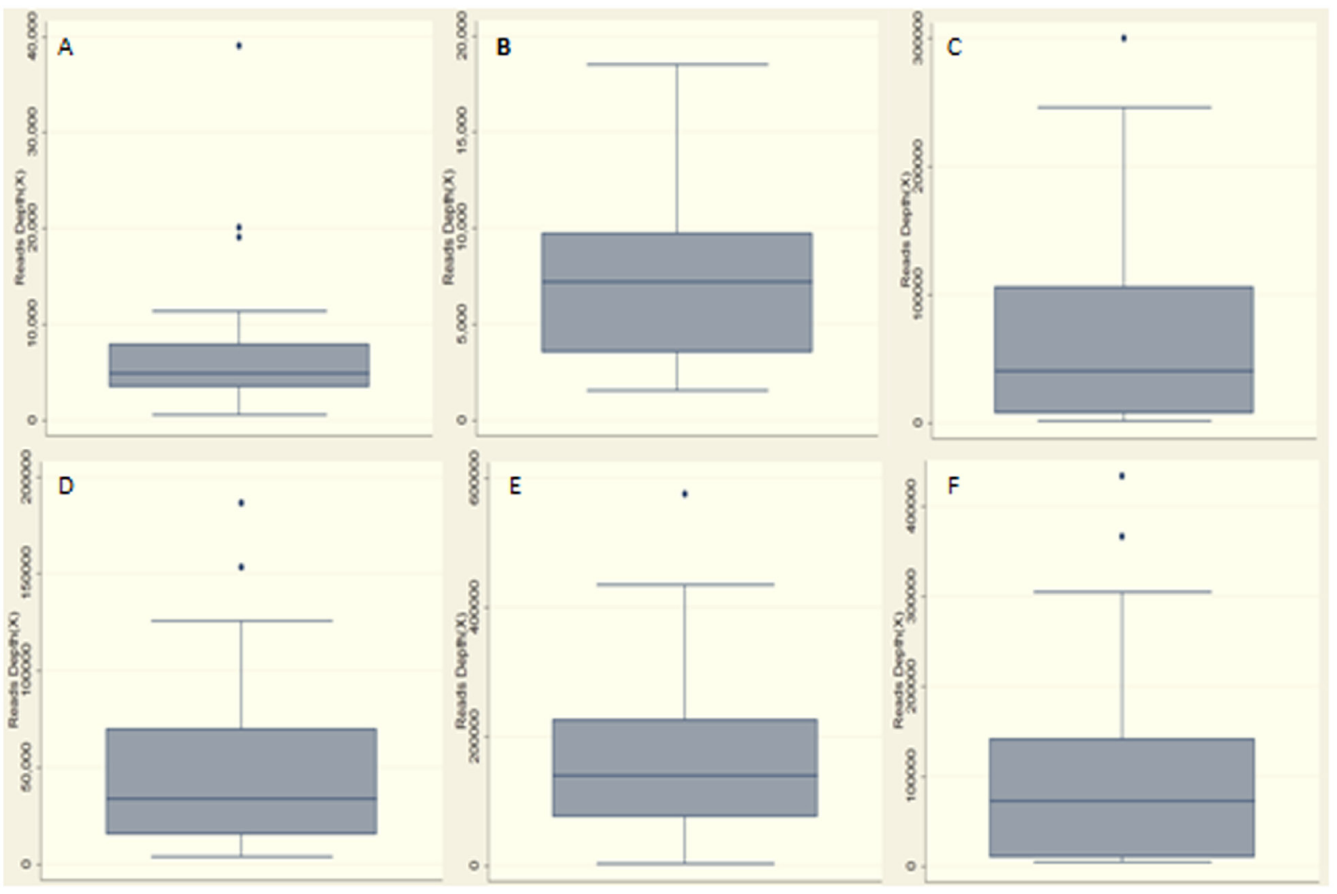
Statistics of read depth for Miseq sequence in plasma and matched tumor DNA samples of the patients **A.** Read depth of *TERT* gene in tumor DNA samples; **B.** Read depth of *CTNNB1* gene in tumor DNA samples; **C.** Read depth of *TP53* gene in tumor DNA samples; median read depth in tumor samples was 7,194× and the average was 27,410×; **D.** Read depth of *TERT* gene in plasma DNA samples; **E.** Read depth of *CTNNB1* gene in plasma DNA samples; **F.** Read depth of *TP53* gene in plasma DNA samples; median read depth in plasma samples was 72,184× and the average was 106,000×.

The relevant mutation sites that we designed were detected in DNA samples from plasma and matched tumor tissue. The sequence depth of the targeted regions is shown in Figure 1. We detected and captured ctDNA with HCC-associated mutations in eight patients (19.5%). Among these, genetic mutations were detected in plasma samples of two patients (4.9%) for the *TERT* genetic mutation, four (9.8%) patients for *CTNNB1* mutation, and two (4.9%) patients for the *TP53* mutation. Interestingly, one patient (HCC32) had a tumor-associated mutation of HCC (c.122C>T, CTNNB1) in plasma DNA but not in the primary tumor DNA. This is because ctDNA fragments are collected from all tumors in a patient's body and could therefore overcome the limitation of tumor heterogeneity that limits traditional tissue biopsy. We also performed the same analysis in the control group and found that only one plasma sample had the corresponding *TERT* mutation. The specificity of this analysis was 90%. Detailed results are presented in Supplementary Tables 2 and 3.

Furthermore, we randomly selected five patient plasma samples and performed repeated trials. The sequence depth of the targeted regions increased but the results were the same (Supplementary Table 4). We also used Sanger sequencing to detect these putative somatic mutations in genomic DNA of these 41 patients, with negative results in all cases (data not shown). Therefore, the somatic genetic alterations that we selected for analysis were specific for HCC.

### Correlation between detectable somatic mutations in plasma and clinicopathologic characteristics

We analyzed whether there was a relationship between somatic mutation status in plasma and the patients' clinicopathologic characteristics and found that corresponding somatic mutations in plasma DNA correlated with vascular invasion. Specifically, there was a significantly higher probability of capturing and detecting ctDNA with tumor-associated mutation status when the HCC patient suffered vascular invasion (P=0.041 for plasma DNA; Table 1). Other parameters, such as cirrhosis, and tumor size, did not significantly affect the detection rate of ctDNA. Similarly, we also found that the mutation status in primary tumor DNA did not correlate with patient age or other clinicopathologic characteristics. The results of these analyses are shown in Table 1.

### Correlation between detectable somatic mutation in plasma and survival

**Figure 2:**
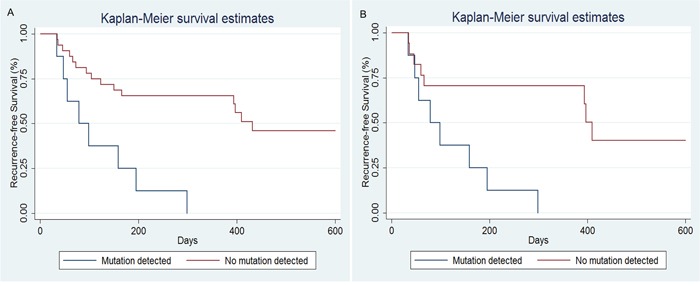
Recurrence-free survival (RFS) curves for HCC patients included in our study **A.** Recurrence-free survival in subjects with detectable versus undetectable tumor-associated mutations in plasma DNA. This analyses revealed that patients with detectable tumor-associated somatic mutations in circulating cfDNA were more likely to relapse than those in whom the detection was negative (P<0.001, log-rank test). **B.** Similar analyses were performed in patients who suffered vascular invasion, revealing a significant association between tumor-associated somatic mutation status and RFS (P=0.003, log-rank test).

We also investigated whether the status of tumor-associated somatic mutation in plasma could be used to predict clinical outcome in the patients with HCC who received surgical treatment. The follow-up time was from the date of operation until the date of tumor recurrence, or for up to 600 days. The data were adjusted from 41 to 40 because one of the patients (HCC01) without corresponding mutations present in plasma DNA was lost to follow-up. The median recurrence-free survival time for patients with tumor-associated somatic mutations detected in plasma DNA was 89 days (range, 34 to 299 days), compared with 365 days (range, 36 to 600 days) for patients with no mutation in plasma. Our data revealed that patients with detectable tumor-associated mutations in circulating cfDNA were more likely to relapse than those in whom the corresponding sequence alteration was undetectable (P<0.001, log-rank test, Figure 2A). Similar results were obtained when we stratified the data by mutation status just in patients who suffered vascular invasion (P=0.003, log-rank test, Figure 2B). In addition, 14 patients were not suffered tumor recurrence in the follow-up period, and all of them had negative ctDNA analyses. So, the specificity of prognosis in our study is 100%.

### Correlation between detectable somatic mutation and concentration of circulating cfDNA

**Figure 3:**
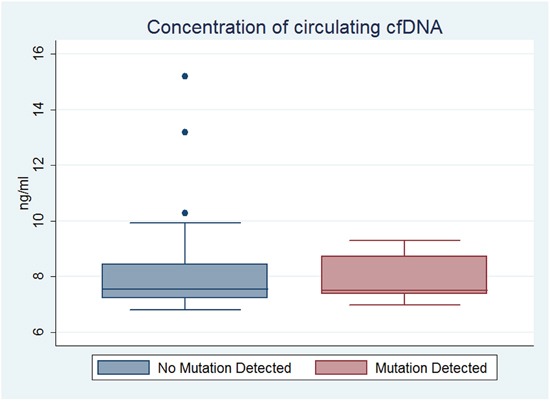
Concentration of circulating cfDNA in subjects with detectable versus undetectable tumor-associated mutations in plasma DNA The median, 25th and 75th percentile, and upper and lower adjacent values of concentration of circulating cfDNA quantified by PicoGreen assay had been shown in box-and-whisker plots. There was no significant correlation between detectable somatic mutation status and the concentration of circulating cfDNA (P=0.818 by Wilcoxon rank sum test).

We also analyzed the relationship between tumor-associated mutations present in plasma and the concentration of circulating cfDNA. We found that the median concentration of circulating cfDNA in patients with tumor-associated mutation status in plasma was 7.501 ng/ml (range, 6.973 to 9.293 ng/ml), compared with 7.540 ng/ml (range, 6.810 to 15.205 ng/ml) for patients with no tumor-associated mutation detected. There was no significant difference between the two groups (Z=−0.230, P=0.818, Figure 3).

## DISCUSSION

The clinical value of using circulating cfDNA as a relatively noninvasive biomarker in cancer has been actively explored. However, we previously performed a meta-analysis to evaluate the use of traditional analyses of circulating cfDNA for HCC diagnosis and found that the results lacked robustness [15]. This is easy to understand because the number of circulating genic fragments that derive from tumor tissue is very small compared with the number of total circulating DNA fragments (<1.0%) [16, 17] and therefore hard to detect by traditional technology.

Our current study used the MiSeq™ System to detect selected mutation fragments in ctDNA from the plasma of HCC patients for the first time, and demonstrated that this approach was suitable as a surrogate tissue for HCC patients.

The results of our study further suggested that mutation analysis of plasma DNA could predict a shorter recurrence-free survival time in HCC patients. In our population, all of the patients with tumor-associated somatic mutation status in ctDNA suffered disease recurrence within 1 year, compared with only 31% of patients without corresponding mutation status. It appeared that the presence of tumor-associated mutations in plasma and disease recurrence had a close relationship, consistent with previous reports [16]. This result may be particularly useful for assessing prognosis and determining subsequent therapy.

In addition, we found that vascular invasion was an important factor influencing the ability to detect ctDNA carrying tumor-associated somatic mutations of HCC. This is easy to explain because vascular invasion will greatly increase the probability of the release of nucleic acid fragments into the circulation. In other words, detectable tumor-associated mutation status in ctDNA might indirectly imply that the patient suffered vascular invasion and might be the reason why “liquid biopsy” could predict the poor prognosis of HCC patients in our study. However, none of the other clinicopathologic characteristics of patients tested affected the probability of detecting ctDNA. Similarly, there was no significant correlation between detectable somatic mutation and the concentration of circulating cfDNA. This is probably because a higher percentage of nucleic acids might be released from non-mutation bearing tissues under various pathologic and normal physiologic statuses.

It is well recognized that most cancers are heterogeneous and that different areas of the same tumor can show different genetic profiles [18]. This might be the major limitation of traditional tissue biopsy [7, 17]. In our study, the fortuitous discovery of one patient in whom the tumor-associated mutation was detected in ctDNA but not in the tumor DNA provided powerful evidence that a “liquid biopsy” could overcome this kind of limitation.

Furthermore, we also showed that the tumor-associated mutations detected in ctDNA could use for HCC diagnosis. However, the sensitivity of this approach was not satisfactory, because these verified hotspots in the *TERT*, *CTNNB1*, and *TP53* genes were found in plasma DNA in only 3.9%, 7.8%, and 3.9% of our patients, respectively, even though careful measures were taken. However, if we combined these genes, the sensitivity could be improved 2- to 4-fold. Tracking multiple mutations might increase the robustness by compensating for effects of mutational drift or sampling noise. Thus, the greater number of mutation fragments in different genes used for detection in plasma, the better diagnostic performance would be got. In our study, the specificity for combined detection was as high as 90%.

Targeted sequencing for ctDNA has previously been applied in some types of tumor, such as pancreatic cancer with a sensitivity of 43% [19]and non-small cell lung cancer with a sensitivity of 34.3% [20]. These results were superior to those that we obtained for HCC. One possible explanation for this discrepancy might be ascribed to the complex immunologic system of the liver. For example, among the innate immune components containing dozens of leukocytes and antigen-presenting cells [21, 22], Kuppffer cells, which reside as stationary macrophages in the liver sinusoids, have been shown to perform functions of trapping, phagocytosing, and elimination [23] even for targets of circulating tumor cells and circulating DNAs [24, 25]. If the concentration of mutation fragments of ctDNA was far less than that of DNA derived from tissues without mutations, our evaluation of plasma DNA might miss such mutations [20]. Another possible explanation might be related to the targeted sequencing that we chose. The sequence changes that were chosen by Sausen et al. covered 116 specific genes in the targeted analyses [19], whereas Bai et al. analyzed *EGFR* mutations in exons 19 and 21 [20]. In our study, targeted sequencing only covered three different regions of the *hTERT*, *CTNNB1*, and *P53* genes with total coverage of 453bp. The small number of mutation fragments used for detection might contribute to the difference in results between our study and previous reports.

Of course, this research is just beginning. We are currently planning a prospective study with multiple targets covering almost all HCC-associated mutations to improve the value of the circulating cfDNA assay in HCC. Once validated in additional clinical trials, our approach would provide advantageous information for molecular assessment in personalized HCC management.

In conclusion, we present a framework for the use of circulating tumor DNA as a liquid biopsy for HCC patients for the first time. The results of our study provided strong evidence that the mutation fragments present in plasma were associated with vascular invasion and might be used for predicting a shorter recurrence-free survival time. We also confirmed that examination of ctDNA for genetic alterations could overcome the limitation of tumor heterogeneity. Moreover, the noninvasive detection of TERT, CTNNB1 and TP53 mutations by circulating cfDNA could be a reliable approach for HCC, and detection of multiple mutations in different genes would improve the diagnostic performance. Prospective validation based upon this initial study is now needed.

## MATERIALS AND METHODS

### Sample collection and DNA extraction

This study was approved by the Ethics Review committee of Peking Union Medical College. HCC patients were eligible if they agreed to undergo a circulating cfDNA assay on plasma obtained before surgery and provided signed informed consent. No more restriction was existed. The peripheral blood was drawn into EDTA tubes and within 1 hour subjected to centrifugation at 800g for 10 min. The plasma was separated from blood cells and subjected to an additional centrifugation step at a high speed of 16,000g for 10 min to remove any remaining cellular debris [26]. The plasma supernatant and matched blood cells were stored separately at −80°C. Corresponding tumor samples, about 1 cm*1 cm, were taken from the central part of the tumor tissue and frozen in liquid nitrogen immediately after surgery, and then were transferred to −80°C for storage. The necrotic tissue was also avoided when tissue samples were obtained. The pathologic diagnosis of tumor was based on histologic criteria [27].

DNA was extracted from frozen tissue and matched blood cells using the QIAamp DNA mini kit (Qiagen Co. Ltd, DE). Circulating cfDNA was extracted from 720μl of plasma per sample using the NucleoSpin Plasma XS kit (Macherey & Nagel GmbH & Co. KG, DE) strictly following the manufacturer's instructions.

### Study design and MiSeq sequencing

**Figure 4:**
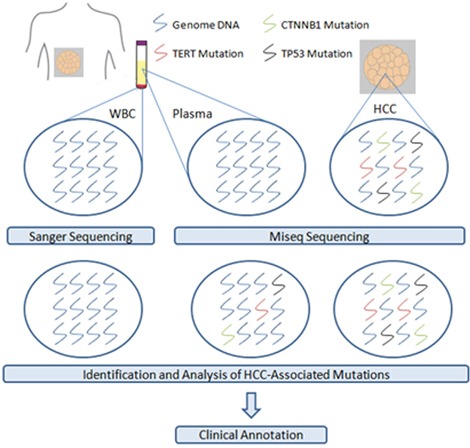
Identification and analysis of HCC-associated mutations from plasma and tumor samples by Miseq sequencing Overview of the study design: DNA samples were extracted from plasma, white blood cells (WBC) and matched tumor tissue from hepatocellular carcinoma (HCC) patients respectively. Using the MiSeq™ sequencing, plasma and matched tumor DNA samples were analyzed for hotspot mutations in the *TERT*, *CTNNB1*, and TP53 genes that had been verified as the most prevalent mutations in HCC. These HCC-associated genetic mutations could be detected in ctDNA and this was evaluated for potential clinical utility or as prognostic indicators. Sanger sequencing was performed for genome DNA from white blood cells and used to verify these somatic genetic alterations that we selected for analysis were specific for HCC.

Some hotspots in the *TERT*, *TP53*, and *CTNNB1* genes have emerged as the most prevalent sites of mutation in HCC patients, such as −124G>A in *TERT*, c.747G>T in *TP53* and c.134C>T in *CTNNB1*[13, 28]. In theory, these specific genetic aberrations would be present in ctDNA and shed into the bloodstream. This kind of actionable information could be exploited in a liquid biopsy for clinical and investigational applications (Figure 4). Therefore, we designed primers to amplify different regions containing frequent mutations of these three genes in tumor tissue and plasma (Supplementary Table 5). The following process was independently and participants were blinded to the samples and patient's clinical characteristics. All of the amplification products were less than 170bp in length. Each PCR reaction contained 0.3μl primer STAR GXL DNA polymerase (Takara Bio Company, Japan), 3 μl of 5×primer STAR GXL Buffer (Takara), 5 μM of each primer, 2.5 mM of dNTP mixture, 1.5 μl template DNA purified from tissue samples or 5 μl template DNA purified from plasma, and ddH_2_O to give a total volume of 15 μl. The PCR conditions were 95°C for 2 minutes; 35 cycles of 98°C for 30 seconds, 60°C for 30 seconds, and 68°C for 30 seconds; with a final incubation at 68°C for 5 minutes. The PCR products were initially identified by Sanger sequencing and sequenced using the MiSeq™ System (Illumina, Inc, US) with Miseq Reagent Kit V3 strictly according to the manufacturer's instructions.

The MiSeq™ System can perform base calling using integrated Real Time Analysis and produces information about alignment, structural variants, and contig assemblies for each sample. The raw data that were generated from containing base calls per cycle needed to be trimmed by the software Mothur [29] with the conditions of minimum Phred quality score of 20 and maximum homopolymer of 10. Furthermore, ambiguous bases and selfsame barcodes and primers were also abandoned. Finally, a total of 16,409,410 paired-end reads were retained for further processing. We used Burrows Wheeler Aligner's Smith-Waterman Alignment [30] to align all of these qualified sequencing reads to a reference genome (H19) and software of VarScan [31] to detect single nucleotide variants or indels with high sensitivity and specificity. We also used the ANNOVAR tool [32] to annotate genetic variations among these high-throughput sequencing data.

### Sanger sequencing

We used Sanger sequencing to verify the PCR products before MiSeq sequencing and to identify mutations in genomic DNA isolated from blood cells. The PCR products were sequenced using ABI 3730 (Applied Biosystems Inc, US). Mutation Surveyor Software [33] was used for mutational analysis.

### Quantification of circulating cfDNA from plasma

We quantified the amount of total circulating cfDNA using Quant-iT™ PicoGreen^®^ dsDNA Kit (Thermo Fisher Scientific, US). This kind of ultrasensitive fluorescent nucleic acid stain is very sensitivity for DNA quantification. Each DNA sample was diluted with Tris-EDTA buffer (10 mM Tris-HCL, 1 mM EDTA, pH 7.5) to 100 μl in 96-well microplate, and then 100 μl of Quant-iT™ PicoGreen^®^ reagent (200-fold dilution) was added to each cuvette with a final volume of 200 μl. The mixed working solution was protected from light and incubated for 5 minutes at room temperature. After that, the fluorescent signal of each sample was measured at standard fluorescein wavelengths (excitation was 480 nm and emission was 520 nm) using Synergy H1 (Multi-Mode Reader, BioTek, US). All of samples were assessed in triplicate. Standard curve was generated using lambda DNA standard provided by this kit.

### Statistical analysis

We used Stata software (version 12.0; Stata Corporation LP; College Station, TX) to perform statistical analyses. The X^2^-test and Fisher's exact test was used to assess the relationship between genetic mutation status and each of the clinical and pathologic characteristics. Curves for recurrence-free survival (calculated as the time from operation to tumor recurrence) were constructed using the Kaplan–Meier method and assessed using the log-rank test. We also used the Wilcoxon rank sum test to determine whether the quantity of circulating cfDNA had a significant impact on mutation detection in ctDNA. For each analysis, a result was considered to be statistically significant if the P-value was less than 0.05.

## SUPPLEMENTARY TABLES








