# Supplementary material for: Noninvasive detection of tumor-associated mutations from circulating cell-free DNA in hepatocellular carcinoma patients by targeted deep sequencing
Source: Oncotarget. 2016 May 26;7(26):40481–90. doi: 10.18632/oncotarget.9629 (PMC5130021; doi:10.18632/oncotarget.9629)
Supplement: Supplementary file 1 [file oncotarget-07-40481-s001.pdf]

Noninvasive detection of tumor-associated mutations from circulating cell-free DNA in hepatocellular carcinoma patients by targeted deep sequencing

SUPPLEMENTARY TABLES

Supplementary Table S1: Detailed information of the prior-treatment status of HCC patients

See Supplementary File 1

Supplementary Table S2: Clinical characteristics and mutation status in plasma DNA of control group

Subjects	Age (Year)	Sex	Alcohol intake	Cirrhosis	HBV	Diagnosis	Mutation		
							P-TERT	P-CTNNB1	P-TP53
**Con1**	37	F	No	No	No	Hepatic hemangiomas	-135G>T	Not detected	Not detected
**Con2**	50	M	Yes	No	No	hepatic hemangiomas	Not detected	Not detected	Not detected
**Con3**	36	F	No	No	No	Hepatic EHE	Not detected	c.90C>T	Not detected
**Con4**	65	M	Yes	No	No	FNH	-166C>T	131C>T	Not detected
**Con5**	33	M	Yes	No	No	HCE	Not detected	120T>C	Not detected
**Con6**	70	M	No	No	No	ICC	-189A>G -166C>T	Not detected	Not detected
**Con7**	25	F	No	No	No	HC	-189A>G -124C>T	Not detected	c.730G>T c.729G>A
**Con8**	35	F	No	No	No	HC	Not detected	Not detected	Not detected
**Con9**	25	F	No	No	No	HC	Not detected	Not detected	Not detected
**Con10**	23	F	No	No	No	HC	Not detected	Not detected	Not detected

Abbreviations: F, Female; M, Male; Con, Control group; Hepatic EHE, Hepatic epithelioid hemangioendothelioma; FNH, Focal nodular hyperplasia; HCE, Hepatic cystic echinococcosis; ICC, Intrahepatic cholangiocellular carcinomas; HC, Health control.

Supplementary Table S3: Variants in TERT, CTNNB1 and TP53 detected in tumor tissue and plasma of HCC patients

See Supplementary File 2

Supplementary Table S4: Repeated trials in five randomly selected patients

Subject	Mutations			Mutations (repeated trials)		
	P-TERT	P-CTNNB1	P-TP53	P-TERT	P-CTNNB1	P-TP53
**HCC06**	-168A>C	Not detected	Not detected	Not detected	Not detected	Not detected
**HCC07**	-166C>T	Not detected	Not detected	-113C>T<br>-104T>C	Not detected	c.737T>C
**HCC09**	-189A>G	Not detected	Not detected	-189A>T<br>-113C>T<br>-104T>C<br>-98T>C	Not detected	c.737T>C
**HCC16**	-168A>G	c.116C>T	Not detected	-168A>G	Not detected	Not detected
**HCC38**	-124C>T	Not detected	c.737T>A	-189A>T<br>-124C>T<br>-113C>T<br>-104T>C	Not detected	c.737T>A

Abbreviations: Reads Depth in repeated trials had been increased (Median: 92461X; Average: 156810X).

Supplementary Table S5: Primer design for MiSeq sequencing

See Supplementary File 3
