# Supplementary material for: Noninvasive detection of tumor-associated mutations from circulating cell-free DNA in hepatocellular carcinoma patients by targeted deep sequencing
Source: Oncotarget. 2016 May 26;7(26):40481–90. doi: 10.18632/oncotarget.9629 (PMC5130021; doi:10.18632/oncotarget.9629)
Supplement: Supplementary file 2 [file oncotarget-07-40481-s002.docx]

**Supplementary Table 1: Detailed information of the prior-treatment status of HCC patients.**

| Subjects | Age  (Year) | HBV/HCV | Alcohol | Cirrhosis | AFP  (ng/ml) | ALT  (U/L) | AST  (U/L) | Albumin  (g/L) | TBIL  (umol/L) | PT  (s) | Ascites | C-P Class | Surgery Only |
| --- | --- | --- | --- | --- | --- | --- | --- | --- | --- | --- | --- | --- | --- |
| HCC01 | 67 | HBV | Y | N | 9.3 | 26 | 42 | 41 | 12.7 | 11.8 | N | A | Y |
| HCC02 | 49 | HBV | Y | Y | 90.4 | 53 | 40 | 45 | 10.7 | 12.4 | N | A | Y |
| HCC03 | 44 | HBV | Y | N | 2.6 | 26 | 29 | 43 | 34.2 | 13.5 | N | A | Y |
| HCC04 | 56 | HBV | N | Y | 4.2 | 23 | 24 | 41 | 13.4 | 13.0 | N | A | Y |
| HCC05 | 53 | HBV | Y | Y | 60500 | 27 | 31 | 42 | 15.2 | 12.1 | N | A | Y |
| HCC06 | 56 | HBV | N | Y | 66.5 | 24 | 37 | 39 | 16.3 | 11.9 | N | A | Y |
| HCC07 | 48 | HBV | Y | N | 648.7 | 31 | 45 | 44 | 14.5 | 11.8 | N | A | Y |
| HCC08 | 61 | HBV | N | Y | 1458 | 32 | 41 | 42 | 15.1 | 12.6 | N | A | Y |
| HCC09 | 54 | HBV | N | Y | 60500 | 52 | 77 | 40 | 17.9 | 11.2 | Y | A | Y |
| HCC10 | 61 | HBV | N | N | 6.7 | 25 | 24 | 40 | 9.9 | 11.7 | N | A | Y |
| HCC11 | 47 | NA | Y | Y | 9.2 | 36 | 27 | 38 | 11.2 | 13.0 | N | A | Y |
| HCC12 | 61 | NA | N | Y | 1.9 | 31 | 42 | 39 | 17.8 | 12.2 | N | A | Y |
| HCC13 | 51 | HBV | N | N | 19803 | 52 | 83 | 34 | 12.1 | 11.8 | N | A | Y |
| HCC14 | 50 | HBV | N | Y | 5764 | 25 | 28 | 41 | 16.1 | 12.0 | N | A | Y |
| HCC15 | 76 | HBV | N | N | 746.6 | 21 | 26 | 39 | 14.1 | 12.0 | N | A | Y |
| HCC16 | 68 | HBV | N | Y | 0.9 | 12 | 18 | 47 | 9.7 | 11.4 | N | A | Y |
| HCC17 | 26 | HBV | Y | Y | 14378 | 17 | 21 | 43 | 16.0 | 12.2 | N | A | Y |
| HCC18 | 72 | HBV | Y | N | 3.3 | 22 | 27 | 48 | 13.8 | 10.9 | N | A | Y |
| HCC19 | 39 | HBV | N | N | 11938 | 41 | 27 | 37 | 19.3 | 13.1 | N | A | Y |
| HCC20 | 51 | HBV | N | Y | 2431 | 62 | 53 | 45 | 10.0 | 11.4 | N | A | Y |
| HCC21 | 60 | HBV | N | N | 42701 | 28 | 28 | 45 | 27.2 | 10.2 | N | A | Y |
| HCC22 | 58 | HBV | Y | Y | 325.4 | 28 | 38 | 43 | 12.1 | 11.7 | N | A | Y |
| HCC23 | 50 | HBV | Y | Y | 142.3 | 104 | 82 | 43 | 15.6 | 11.8 | N | A | Y |
| HCC24 | 67 | HBV | N | Y | 1111 | 39 | 36 | 41 | 18.2 | 12.1 | N | A | Y |
| HCC25 | 62 | HBV | Y | N | 4075 | 27 | 26 | 46 | 15.6 | 12.5 | N | A | Y |
| HCC26 | 57 | HBV | N | Y | 183.5 | 17 | 25 | 42 | 14.4 | 11.4 | N | A | Y |

**Supplementary Table 1: Detailed information of the prior-treatment status of HCC patients (Continued).**

| Subjects | Age  (Year) | HBV/HCV | Alcohol | Cirrhosis | AFP  (ng/ml) | ALT  (U/L) | AST  (U/L) | Albumin  (g/L) | TBIL  (umol/L) | PT  (s) | Ascites | C-P Class | Surgery Only |
| --- | --- | --- | --- | --- | --- | --- | --- | --- | --- | --- | --- | --- | --- |
| HCC27 | 44 | HBV | N | Y | 3627 | 34 | 29 | 46 | 15.5 | 11.8 | N | A | Y |
| HCC28 | 32 | NA | N | N | 105.1 | 27 | 23 | 45 | 17.3 | 12.0 | N | A | Y |
| HCC29 | 36 | HBV | N | N | 9250 | 336 | 187 | 41 | 22.4 | 13.5 | N | A | Y |
| HCC30 | 49 | HBV | Y | Y | 17.5 | 114 | 80 | 41 | 8.5 | 11.7 | N | A | Y |
| HCC31 | 39 | HBV | N | Y | 1.7 | 23 | 18 | 49 | 10.5 | 10.2 | N | A | Y |
| HCC32 | 62 | HBV | N | Y | 6.5 | 26 | 30 | 44 | 15.8 | 11.7 | N | A | Y |
| HCC33 | 73 | HBV | N | N | 4.69 | 21 | 42 | 42 | 13.7 | 12.4 | N | A | Y |
| HCC34 | 78 | HBV | N | N | 575.3 | 28 | 29 | 34 | 6.8 | 10.5 | Y | B | Y |
| HCC35 | 75 | HBV | Y | Y | 6.6 | 25 | 31 | 38 | 11.2 | 11.9 | N | A | Y |
| HCC36 | 50 | HBV | N | Y | 396.7 | 35 | 46 | 48 | 28.1 | 13.1 | N | A | Y |
| HCC37 | 66 | HBV | N | N | 5.8 | 22 | 36 | 41 | 23.1 | 12.3 | N | A | Y |
| HCC38 | 63 | HBV | N | Y | 444.4 | 21 | 66 | 41 | 12.8 | 13.2 | N | A | Y |
| HCC39 | 60 | HBV | Y | N | 2664 | 30 | 41 | 45 | 12.2 | 11.8 | N | A | Y |
| HCC40 | 54 | HBV | N | N | 2.6 | 22 | 24 | 41 | 16.4 | 11.7 | N | A | Y |
| HCC41 | 62 | HBV | N | Y | 11278 | 31 | 29 | 40 | 14.4 | 12.7 | N | A | Y |

Abbreviations: HBV, hepatitis B virus; HCV, hepatitis C virus; AFP, alpha-fetoprotein; ALT, alanine aminotransferase; AST, aspartate aminotransferase; TBIL: total bilirubin; PT: prothrombin time; C-P Class: Child-Pugh Class; Y: Yes; N, No;
