# Supplementary material for: Noninvasive detection of tumor-associated mutations from circulating cell-free DNA in hepatocellular carcinoma patients by targeted deep sequencing
Source: Oncotarget. 2016 May 26;7(26):40481–90. doi: 10.18632/oncotarget.9629 (PMC5130021; doi:10.18632/oncotarget.9629)
Supplement: Supplementary file 3 [file oncotarget-07-40481-s003.docx]

**Supplementary Table 3: Variants in TERT, CTNNB1 and TP53 detected in tumor tissue and plasma of HCC patients.**

| Subjects | T-TERT | | P-TERT | | T-CTNNB1 | | P-CTNNB1 | | T-TP53 | | P-TP53 | |
| --- | --- | --- | --- | --- | --- | --- | --- | --- | --- | --- | --- | --- |
|  | **mutation** | **frequency** | **mutation** | **frequency** | **mutation** | **frequency** | **mutation** | **frequency** | **mutation** | **frequency** | **mutation** | **frequency** |
| HCC01 | -189A>G  -186T>C  -168A>C  -124C>T | 1.82%  1.68%  1.79%  42.10% | Not detected | N/A | Not detected | N/A | Not detected | N/A | c.747G>T | 3.82% | Not detected | N/A |
| HCC02 | -124C>T | 7.38% | Not detected | N/A | Not detected | N/A | Not detected | N/A | c.747G>T | 2.35% | c.747G>T | 1.06% |
| HCC03 | -124C>T | 56.28% | Not detected | N/A | c.100G>A | 1.39% | Not detected | N/A | c.747G>T | 1.74% | Not detected | N/A |
| HCC04 | -124C>T | 5.58% | Not detected | N/A | Not detected | N/A | Not detected | N/A | c.747G>T | 2.59% | Not detected | N/A |
| HCC05 | -189A>C  -186A>C  -124C>T | 2.06%  1.99%  5.44% | Not detected | N/A | Not detected | N/A | Not detected | N/A | c.747G>T | 3.40% | Not detected | N/A |
| HCC06 | -124C>T | 7.32% | -168A>C | 1.4% | Not detected | N/A | Not detected | N/A | c.747G>T | 2.11% | Not detected | N/A |
| HCC07 | -124C>T  -113C>T  -104T>C  -98T>C  -96T>C | 5.23%  2.06%  9.95%  2.14%  1.61 | -166C>T | 1.27% | Not detected | N/A | Not detected | N/A | c.729G>A  c.730G>T  c.737T>C | 2.20%  2.26%  3.37% | Not detected | N/A |
| HCC08 | Not detected | N/A | Not detected | N/A | Not detected | N/A | Not detected | N/A | c.747G>T | 2.54% | Not detected | N/A |

**Supplementary Table 3: Variants in TERT, CTNNB1 and TP53 detected in tumor tissue and plasma of HCC patients (Continued).**

| Subjects | T-TERT | | P-TERT | | T-CTNNB1 | | P-CTNNB1 | | T-TP53 | | P-TP53 | |
| --- | --- | --- | --- | --- | --- | --- | --- | --- | --- | --- | --- | --- |
|  | **mutation** | **frequency** | **mutation** | **frequency** | **mutation** | **frequency** | **mutation** | **frequency** | **mutation** | **frequency** | **mutation** | **frequency** |
| HCC09 | -124C>T  -113C>T | 5.52%  13.25% | -189A>G | 6.65% | Not detected | N/A | Not detected | N/A | c.729G>A  c.730G>T  c.737T>C | 4.17%  4.20%  3.13% | Not detected | N/A |
| HCC10 | Not detected | N/A | Not detected | N/A | Not detected | N/A | Not detected | N/A | Not detected | N/A | Not detected | N/A |
| HCC11 | Not detected | N/A | Not detected | N/A | c.133T>G | 1.19% | Not detected | N/A | Not detected | N/A | Not detected | N/A |
| HCC12 | Not detected | N/A | Not detected | N/A | c.133T>G | 35.59% | Not detected | N/A | Not detected | N/A | Not detected | N/A |
| HCC13 | -186T>C  -124C>T | 1.24%  3.82% | Not detected | N/A | Not detected | N/A | Not detected | N/A | Not detected | N/A | Not detected | N/A |
| HCC14 | Not detected | N/A | Not detected | N/A | C134C>T | 28.29% | C134C>T | 2.31% | Not detected | N/A | Not detected | N/A |
| HCC15 | -124C>T | 16.71% | -168A>C | 2.19% | Not detected | N/A | Not detected | N/A | Not detected | N/A | Not detected | N/A |
| HCC16 | -171C>T | 1.41% | -168A>G | 1.06% | c.107A>C | 33.99% | c.116C>T | 1.4% | Not detected | N/A | Not detected | N/A |
| HCC17 | Not detected | N/A | Not detected | N/A | Not detected | N/A | Not detected | N/A | Not detected | N/A | Not detected | N/A |
| HCC18 | -189A>C  -168A>C  -124C>T | 1.66%  1.67%  10.58% | Not detected | N/A | c.121A>G | 47.03% | c.121A>G | 1.16% | c.747G>T | 2.69% | Not detected | N/A |

**Supplementary Table 3: Variants in TERT, CTNNB1 and TP53 detected in tumor tissue and plasma of HCC patients (Continued).**

| Subjects | T-TERT | | P-TERT | | T-CTNNB1 | | P-CTNNB1 | | T-TP53 | | P-TP53 | |
| --- | --- | --- | --- | --- | --- | --- | --- | --- | --- | --- | --- | --- |
|  | **mutation** | **frequency** | **mutation** | **frequency** | **mutation** | **frequency** | **mutation** | **frequency** | **mutation** | **frequency** | **mutation** | **frequency** |
| HCC19 | -124C>T | 7.79% | Not detected | N/A | c.101G>T | 27.28% | c.101G>T | 3.46% | c.747G>T | 2.39% | Not detected | N/A |
| HCC20 | -124C>T | 5.73% | Not detected | N/A | c.100G>C  c.121A>G | 1.10%  1.31% | Not detected | N/A | c.747G>T | 3.03% | Not detected | N/A |
| HCC21 | -124C>T | 36.86% | Not detected | N/A | Not detected | N/A | Not detected | N/A | c.747G>T | 3.33% | Not detected | N/A |
| HCC22 | -124C>T | 6.40% | Not detected | N/A | Not detected | N/A | Not detected | N/A | c.747G>T | 56.52% | c.747G>T | 6.01% |
| HCC23 | -168A>C  -124C>T | 1.38%  52.12% | Not detected | N/A | c.100G>C | 27.98% | c.74G>T | 1.05% | c.747G>T | 3.28% | Not detected | N/A |
| HCC24 | -120G>C | 1.05% | Not detected | N/A | c.100G>C  c.121A>G | 1.43%  1.15% | Not detected | N/A | c.747G>T | 2.44% | Not detected | N/A |
| HCC25 | -124C>T | 44.86% | Not detected | N/A | Not detected | N/A | c.74G>T | 1.23% | c.747G>T | 1.51% | Not detected | N/A |
| HCC26 | -124C>T  -113C>T  -104T>C  -98T>C | 43.46%  3.81%  5.85%  1.19% | -124C>T | 25.31% | Not detected | N/A | Not detected | N/A | c.729G>A  c.730G>T  c.737T>C | 65.69%  66%  3.17% | c.737T>C | 1.34% |
| HCC27 | -189A>C  -124C>T | 1.07%  5.57% | Not detected | N/A | Not detected | N/A | Not detected | N/A | c.747G>T | 1.49% | Not detected | N/A |
| HCC28 | -124C>T | 2.74% | Not detected | N/A | Not detected | N/A | Not detected | N/A | c.747G>T | 2.04% | Not detected | N/A |

**Supplementary Table 3: Variants in TERT, CTNNB1 and TP53 detected in tumor tissue and plasma of HCC patients (Continued).**

| Subjects | T-TERT | | P-TERT | | T-CTNNB1 | | P-CTNNB1 | | T-TP53 | | P-TP53 | |
| --- | --- | --- | --- | --- | --- | --- | --- | --- | --- | --- | --- | --- |
|  | **mutation** | **frequency** | **mutation** | **frequency** | **mutation** | **frequency** | **mutation** | **frequency** | **mutation** | **frequency** | **mutation** | **frequency** |
| HCC29 | Not detected | N/A | Not detected | N/A | Not detected | N/A | Not detected | N/A | c.747G>T | 2.17% | Not detected | N/A |
| HCC30 | Not detected | N/A | Not detected | N/A | Not detected | N/A | Not detected | N/A | c.747G>T | 2.27% | Not detected | N/A |
| HCC31 | -189A>C  -168A>C  -124C>T | 1.26%  2.10%  3.32% | Not detected | N/A | Not detected | N/A | c.165G>T | 1.15% | c.747G>T | 52.73% | Not detected | N/A |
| HCC32 | Not detected | N/A | Not detected | N/A | Not detected | N/A | c.122C>T | 1.20% | c.747G>T | 2.26% | Not detected | N/A |
| HCC33 | -124C>T | 33.31% | -168A>C | 1.42% | Not detected | N/A | Not detected | N/A | c.747G>T | 1.92% | Not detected | N/A |
| HCC34 | -124C>T | 30.78% | Not detected | N/A | Not detected | N/A | Not detected | N/A | c.747G>T | 2.08% | Not detected | N/A |
| HCC35 | -124C>T | 4.19% | Not detected | N/A | Not detected | N/A | Not detected | N/A | c.747G>T | 1.51% | Not detected | N/A |
| HCC36 | -124C>T | 7.05% | Not detected | N/A | Not detected | N/A | Not detected | N/A | c.747G>T | 2.42% | Not detected | N/A |
| HCC37 | -124C>T | 55.92% | Not detected | N/A | c.100G>A | 24.93% | Not detected | N/A | c.747G>T | 69.88% | Not detected | N/A |
| HCC38 | -124C>T  -113C>T | 62.95%  2.59% | -124C>T | 7.89% | Not detected | N/A | Not detected | N/A | c.729G>A  c.730G>T  c.737T>A | 3.51%  3.68%  71.11% | c.737T>A | 1.94% |

**Supplementary Table 3: Variants in TERT, CTNNB1 and TP53 detected in tumor tissue and plasma of HCC patients (Continued).**

| Subjects | T-TERT | | P-TERT | | T-CTNNB1 | | P-CTNNB1 | | T-TP53 | | P-TP53 | |
| --- | --- | --- | --- | --- | --- | --- | --- | --- | --- | --- | --- | --- |
|  | **mutation** | **frequency** | **mutation** | **frequency** | **mutation** | **frequency** | **mutation** | **frequency** | **mutation** | **frequency** | **mutation** | **frequency** |
| HCC39 | -168A>C  -124C>T | 2.37%  8.85% | Not detected | N/A | Not detected | N/A | Not detected | N/A | Not detected | N/A | Not detected | N/A |
| HCC40 | -124C>T  -113C>T | 34.06%  7.16% | -189A>G | 1.02% | Not detected | N/A | Not detected | N/A | c.729G>A  c.737T>C | 2.88%  2.93% | Not detected | N/A |
| HCC41 | Not detected | N/A | Not detected | N/A | Not detected | N/A | Not detected | N/A | c.747G>T | 3.68% | Not detected | N/A |

Abbreviations: TERT, telomerase reverse transcriptase; CTNNB1, cadherin-associated protein, beta 1; TP53, tumor protein p53; T-TERT, TERT mutations in tumor tissue DNA; P-TERT, TERT mutations in plasma DNA; T-CTNNB1, CTNNB1 mutations in tumor tissue DNA; P-CTNNB1, CTNNB1 mutations in plasma DNA; T-TP53, TP53 mutations in tumor tissue DNA; P-TP53, TP53 mutations in plasma DNA;
