# Supplementary material for: Noninvasive detection of tumor-associated mutations from circulating cell-free DNA in hepatocellular carcinoma patients by targeted deep sequencing
Source: Oncotarget. 2016 May 26;7(26):40481–90. doi: 10.18632/oncotarget.9629 (PMC5130021; doi:10.18632/oncotarget.9629)
Supplement: Supplementary file 4 [file oncotarget-07-40481-s004.docx]

**Supplementary Table 5: Primer design for MiSeq sequencing:**

| Gene | Chromosome | Exon | Primer Forward | Primer Reverse | Amplify Regions^&^ | Coverage | Covered mutations | Mutation  Type |
| --- | --- | --- | --- | --- | --- | --- | --- | --- |
| TERT | 5 | Promoter | CAGCGCTGCCTGAAACTC | GTCCTGCCCCTTCACCTT | g.1,295,165- g.1,295,327 | 163bp | g.1,295,228G>A; -124G>A*  g.1,295,250G>A; -146G>A | Missense  Missense |
| TP53 | 17 | Exon6 | TGGGCCTGTGTTATCTCCTA | GGCAAGTGGCTCCTGACCT | g.7577438- g.7577586 | 148bp | g.7577534C>A; c.747G>T^#^  g.7577556C>T; c.725G>A  g.7577550C>T; c.731G>A  g.7577568C>A; c.713G>T  g.7577545T>C; c.736A>G  g.7577535C>T; c.746G>A  g.7577536T>C; c.745A>G; | Missense  Missense  Missense  Missense Missense  Missense  Missense |
| CTNNB1 | 3 | Exon3 | CAGAAAAGCGGCTGTTAG | ATACAGGACTTGGGAGGT | g.41266059- g.41266195 | 142bp | g.41266124A>G; c.121A>G^  g.41266098A>G; c.95A>G  g.41266137C>T; c.134C>T  g.41266103G>A; c.100G>A  g.41266100T>C; c.97T>C  g.41266136T>C; c.133T>C  g.41266104G>T; c.101G>T  g.41266101C>T; c.98C>T  g.41266110A>C; c.107A>C  g.41266107T>G; c.104T>G  g.41266113C>G; c.110C>G  g.41266125C>A; c.122C>T  g.41266097G>C; c.94G>C | Missense  Missense  Missense  Missense  Missense  Missense  Missense  Missense  Missense  Missense  Missense  Missense  Missense |

&: Hg19

*: Hg19, distance from the ATG start site;

#: Hg19, NM_000546.4;

^: Hg19, NM_001098209.1
